# Accurate Simulations of Lipid Monolayers Require a Water Model with Correct Surface Tension

**DOI:** 10.1021/acs.jctc.1c00951

**Published:** 2022-02-08

**Authors:** Carmelo Tempra, O. H. Samuli Ollila, Matti Javanainen

**Affiliations:** † Institute of Organic Chemistry and Biochemistry, Czech Academy of Sciences, Flemingovo nám. 542/2, 160 00 Prague 6, Czech Republic; ‡ Institute of Biotechnology, University of Helsinki, 00790 Helsinki, Finland

## Abstract

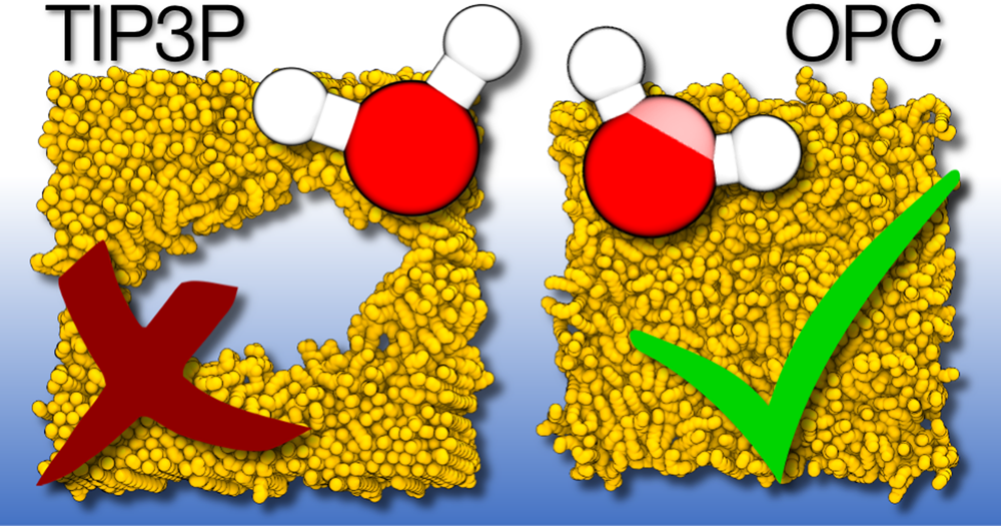

Lipid monolayers provide our lungs and eyes their functionality and serve as proxy systems in biomembrane research. Therefore, lipid monolayers have been studied intensively including using molecular dynamics simulations, which are able to probe their lateral structure and interactions with, e.g., pharmaceuticals or nanoparticles. However, such simulations have struggled in describing the forces at the air–water interface. Particularly, the surface tension of water and long-range van der Waals interactions have been considered critical, but their importance in monolayer simulations has been evaluated only separately. Here, we combine the recent C36/LJ-PME lipid force field that includes long-range van der Waals forces with water models that reproduce experimental surface tensions to elucidate the importance of these contributions in monolayer simulations. Our results suggest that a water model with correct surface tension is necessary to reproduce experimental surface pressure–area isotherms and monolayer phase behavior. The latter includes the liquid expanded and liquid condensed phases, their coexistence, and the opening of pores at the correct area per lipid upon expansion. Despite these improvements of the C36/LJ-PME with certain water models, the standard cutoff-based CHARMM36 lipid model with the 4-point OPC water model still provides the best agreement with experiments. Our results emphasize the importance of using high-quality water models in applications and parameter development in molecular dynamics simulations of biomolecules.

## 1 Introduction

Monolayers of amphiphilic lipids serve as a proxy for lipid membranes in studies of membrane structure and membrane–protein interactions^1,2^ since they are significantly more straightforward to study with a wide range of spectroscopic and microscopic methods as compared to lipid bilayers.^3,4^ Moreover, lipid monolayers form functionally essential structures that line the alveoli in the lungs and cover the surfaces of the eyes.^5^ In the lungs, a pulmonary surfactant (PS) monolayer covers the alveolar liquid, preventing the collapse of the alveoli during exhalation. In the eyes, a tear film lipid layer (TFLL) consists of a monolayer that separates the tear fluid from the nonpolar wax layer of the TFLL, thus helping the latter spread rapidly between eye blinks. Both PS and TFLL are compositionally complex, likely to optimize their mechanical behavior under dynamic conditions. Notably, this behavior depends on the subtle balance of forces at the liquid–air interface.

A Langmuir trough enables the measurement of lipid monolayer surface tension as a function of its area, thereby providing insights into the behavior of PS and TFLL. Moreover, monitoring the changes in the resulting surface pressure–area isotherms upon the addition of biomolecules—such as proteins and drugs—into the aqueous subphase can be used to understand their binding to membranes. Above the main transition temperature (*T*_m_) of the phospholipid, the monolayer remains in the fluidlike liquid expanded (L_e_) phase over a large range of areas. Below the *T*_m_ value, the L_e_ phase transforms upon compression to a gel-like liquid condensed (L_c_) phase through a coexistence plateau.^6^ At very large areas, the pores form in the monolayer, and a gas–L_e_ coexistence appears at very low surface pressures close to 0 mN/m.

Due to their physiological importance, monolayers modeling PS or TFLL have been subjected to numerous computational studies which have utilized both coarse-grained and atomistic molecular dynamics (MD) simulation approaches.^7,8^ MD simulations are also used to complement spectroscopic monolayer experiments, for example, to understand ion binding to membranes.^9^ However, MD simulations have struggled to correctly capture the interactions at interfaces between polar and nonpolar environments that provide PS and TFLL their functionality,^10,11^ yet these interactions need to be properly balanced to reproduce experimental pressure–area isotherms.^12−14^ This discrepancy has been suggested to arise from an underestimated water–air surface tension of common water models^14,15^ and the truncation of long-range van der Waals interactions which compromises the description of the acyl chain–vacuum interface.^13,16,17^

We have recently demonstrated that the CHARMM36 (“C36” from now on) lipid model^13^ combined with the 4-point OPC water model (“OPC4” from now on)^18^ provides nearly quantitative agreement with experimental surface pressure–area isotherms of both single-component^15^ and multicomponent^19^ lipid monolayers. This is because the OPC4 water model reproduces the surface tension of water with a Lennard-Jones (LJ) cutoff of 1.2–1.4 nm. Fortunately, this coincides with the cutoffs recommended to be used with common lipid models such as the C36 (LJ forces switched to zero between 1.0 and 1.2 nm)^13^ and Slipids (strict cutoff for LJ potential at 1.4 nm).^20^ Thus, the OPC4 water model enables more realistic simulations of lipid monolayers without the need to reparameterize the entire lipid model. However, this approach still suffers from issues related to the missing attractive long-range van der Waals forces due to the truncation of the LJ potential. On the other hand, these long-range van der Waals interactions are included in the recent version of the C36 lipid model, coined C36/LJ-PME,^16,17,21^ through a PME-like algorithm.^21−23^ In this model, the glycerol and ester regions of lipids are modified to avoid overcondensation resulting from the increased attraction.^22,24,25^

Our earlier studies^14,15^ suggest that a water model with correct surface tension is necessary to reproduce experimental surface pressure–area isotherms and the phase behavior of lipid monolayers. An inclusion of long-range LJ interactions increases the surface tension of the used CHARMM-specific TIP3P (TIPS3P) water model,^26,27^ albeit not enough for it to match experiments.^28^ The C36/LJ-PME was demonstrated to reproduce the experimental surface tensions at three different areas for a DPPC monolayer,^16,17^ yet its ability to reproduce experimental surface pressure–area isotherms or lipid monolayer phase behavior has not been evaluated.

Here, we aim to understand whether the ability of the water model to reproduce experimental surface tension, the inclusion of long-range van der Waals interactions, or both are critical for the correct description of lipid monolayers in MD simulations. Our results pave the way toward more realistic simulations of lipid monolayers with applications in a wide range of fields from surfactant science to membrane biophysics and pharmacology. The methodological advancement following our results is not limited to only monolayer simulations. Indeed, monolayer surface tensions are used as target parameters in the recently introduced automatic parametrization strategy for C36/LJ-PME,^17^ which is expected to have a wide range of applications for biomolecular simulations of systems with complex compositions.

## 2 Methods

We implemented the C36/LJ-PME model in GROMACS and used it to perform simulations of pure air–water interfaces, lipid bilayers, and lipid monolayers—all with multiple water models. All performed simulations are briefly listed in Table 1. The setup, simulation, and analysis protocols are described in detail in the subsections below (other systems) or in the SI (lipid bilayers). All simulations were performed using GROMACS 2020.^29^ For efficiency and consistency with the CHARMM implementation, all of the LJ-PME simulations performed here with GROMACS used the Lorentz–Berthelot combination rules in the real space and the geometric combination rules in the reciprocal space.^21−23^

**Table 1:** Brief Summary of the Simulations Performed in This Work

system	temperature	purpose
air–water interface 8 × 3 × 5 × 10 ns = 1.2 μs	298, 310, and 323 K	evaluate γ_0_ of 8 different water models with 4 different LJ cutoffs (0.8–1.4 nm) and LJ-PME
POPC bilayers 3 × 5 × 300 ns = 4.5 μs	298, 303, 308, 313, and 318 K	validate our C36/LJ-PME implementation and study its compatibility with 3 water models
DPPC bilayers 4 × 5 × 300 ns = 6.0 μs	323, 328, 333, 338, and 343 K	validate our C36/LJ-PME implementation and study its compatibility with 3 water models; also simulated with standard C36 + TIPS3P
POPC monolayers 3 × 10 × 200 ns = 6.0 μs	298 K	compare C36/LJ-PME with experimental isotherms at 10 areas and with 3 water models
DPPC monolayers 3 × 14 × 300 ns = 12.6 μs	298 K	compare C36/LJ-PME with experimental isotherms at 14 areas and with 3 water models

### 2.1 Implementation of C36/LJ-PME Parameters into GROMACS

We first implemented the “Linkage” versions of the DPPC and POPC C36/LJ-PME models to GROMACS-compatible formats with TopoGromacs^30^ starting from the CHARMM-compatible files downloaded from https://terpconnect.umd.edu/~jbklauda/ff.html. This version of C36/LJ-PME presents minimal changes from the C36 lipid model, and only the nonbonded parameters of the glycerol and ester groups were optimized, along with changes in the respective dihedral parameters. Thus, for both DPPC and POPC, a total of 17 partial charges, 2 Lennard-Jones parameters, and 33 dihedrals differ from their parametrizations in the standard C36 model.^13,16,17^ The modified GROMACS-compatible topology files are available together with all simulation inputs and outputs at DOI 10.5281/zenodo.5720848 (bilayer simulations) and DOI 10.5281/zenodo.5729462 (monolayer simulations).

We validated the parameter conversion by performing identical 300 ns simulations of a DPPC bilayer at 323 K with OpenMM^31^ using C36/LJ-PME parameters in the original (available at DOI: 10.5281/zenodo.5946836) and in the GROMACS-converted formats. These simulations provided essentially identical APL values of 62.5 ± 0.2 and 62.7 ± 0.1 Å^2^, respectively. Single-point energies of a single DPPC lipid from GROMACS and OpenMM with LJ-PME were within 0.02% of each other. As bonded terms were identical between these simulation engines, this small difference likely arises from implementation details of PME and/or LJ-PME.

### 2.2 Surface Tension of Water Models

The surface tensions of eight commonly employed water models were evaluated at different temperatures and with different LJ treatments by simulating the air–water interface.

We first generated a simulation box with 20 052 water molecules and dimensions of 12 × 12 × 4 nm^3^. Next, the shortest box vector was extended to 22 nm in order to create two interfaces between the air (or vacuum) and water. This procedure was repeated for 3-point and 4-point water models. Then, we simulated the systems using various 3-point and 4-point water models: 3-point^32^ (OPC3) and 4-point^18^ Optimal Point Charge (OPC4) models, Simple Point Charge (SPC)^33^ and its extended variant (SPC/E),^34^ three-site Transferrable Intermolecular Potential (TIP3P)^26^ and its CHARMM-variant (TIPS3P),^27^ and four-site Transferrable Intermolecular Potential (TIP4P)^26^ and its updated variant from 2005 (TIP4P/05).^35^

The simulations were performed in constant volume and temperature for 10 ns with varying cutoff values for the Lennard-Jones potential. The simulations used a 2 fs time step. Buffered Verlet lists were used to keep track of atomic neighbors.^36^ Electrostatic interactions were calculated using the smooth Particle Mesh Ewald algorithm.^37,38^ For the Lennard-Jones potential, we used different cutoff values of 0.8, 1.0, 1.2, and 1.4 nm. CHARMM force fields use a switch function for the LJ potential, but this would introduce an extra parameter—the distance at which the switching begins—and thus, we decided to always shift the potential to zero at the cutoff. We applied dispersion corrections^39^ to energy and pressure, as these corrections are used for monolayer simulations with CHARMM. However, the effect of dispersion corrections on the water–air surface tension is within the error estimate.^15^ We also repeated the simulations using LJ-PME.^22,23^ In all simulations, temperature was controlled by the stochastic velocity rescaling algorithm^40^ with a target temperature of either 298, 310, or 323 K and a time constant of 1 ps. The geometry of the water molecules was constrained by the SETTLE algorithm.^41^

The surface tension values were extracted from pressure components normal (*P*_N_) and lateral (*P*_L_) to the interface as
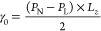
1

Here, , and *P*_*xx*_ = *P*_*yy*_ due to symmetry; the length of the simulation box in the direction normal to the interface is *L*_*z*_. The surface tension values were extracted with the gmx energy command, and the standard error was obtained from block averaging performed by the gmx analyze command. The last 9.9 ns of the 10 ns simulations was used for analysis.

### 2.3 Lipid Monolayer Simulations

A standard setup with two monolayers separated by a slab of water on one side and by a large vacuum space on the other side was used to simulate DPPC and POPC monolayers. The starting structures were taken from our previous work.^15^ The simulations were performed in the canonical ensemble (constant volume, temperature, and particle number) and with periodic boundary conditions in all directions. Monolayers were simulated at different areas per lipid to construct surface pressure–area isotherms that are readily comparable to experiments.

For both DPPC and POPC, simulations were performed with TIPS3P,^27^ the 4-point OPC,^18^ and TIP4/05^35^ water models. These models were chosen as the latter two show the best agreement with experimental water–air surface tension values, whereas the former is the standard water model of the C36/LJ-PME approach. The simulated DPPC monolayers had areas per lipid of 51, 54, 57, 60, 63, 66, 69, 72, 75, 78, 86, 94, 102, and 110 Å^2^ and thus cover the L_c_, L_e_, L_c_/L_e_, and L_e_/gas regions of the experimental isotherms. The POPC monolayers had areas of 58, 64, 70, 78, 86, 94, 102, 110, 118, and 126 Å^2^, covering the L_e_ and L_e_/gas regions. The simulations were either 300 ns (DPPC) or 200 ns (POPC) long, and the first 100 ns was omitted from the analyses, based on the convergence analyses from our recent monolayer work.^15^

The equations of motion were integrated with a leapfrog integrator and with a time step of 2 fs. We used buffered Verlet lists^36^ to keep track of atomic neighbors. The smooth PME^37,38^ and LJ-PME^22,23^ approaches were used to evaluate the long-range electrostatic and van der Waals interactions. The temperatures of the lipid and the solvent were coupled separately to a Nosé–Hoover thermostat^42,43^ with a time constant of 1 ps. P-LINCS^44,45^ was used to constrain bonds involving hydrogen atoms. The geometric combination rules were used for LJ-PME, in line with the CHARMM implementation of C36/LJ-PME.^21^

The surface pressure of the monolayer Π at an area per lipid of APL was extracted from the surface tensions of the pure water–air interface (γ_0_) and the lipid monolayer-coated water–air interface [γ(APL)] as
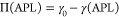
2

The values of γ were extracted using the gmx energy command, and the standard errors were obtained from block averaging performed by the gmx analyze command. The γ_0_ values were taken from the simulations of the pure air–water interface with the corresponding water model. The error of Π was estimated as the sum of the standard errors of the corresponding γ_0_ and γ values.

The phase identity of each lipid was determined by clustering the 10th carbon atoms of the DPPC chains using the DBSCAN algorithm.^46^ A chain was considered to be part of the L_c_ phase, if it had 6 neighbors within 0.71 nm in the plane of the monolayer.

## 3 Results and Discussion

As explained in the Methods section, the conversion of C36/LJ-PME force field parameters from CHARMM to GROMACS format was accurate, but small deviations in nonbonded single point energies between GROMACS and OpenMM probably arise from implementation details of LJ-PME. To evaluate the effect of this on simulations of lipid aggregates, we compared the area per lipid from our GROMACS simulations of DPPC and POPC bilayers at different temperatures with the data from the original C36/LJ-PME publications,^16,17^ our standard C36 simulations, and experiments (Figure S1 in the SI). All simulations consistently give a slightly lower area per molecule than experiments for the DPPC bilayer at 333 K. However, at 323 K, C36/LJ-PME simulated with GROMACS goes into a ripple phase and gives significantly lower area per molecule than when simulated with OpenMM. More condensed membranes and higher melting temperatures have also been previously reported from C36 simulations with GROMACS.^47,48^ We conclude that lipid bilayer APLs in our GROMACS implementation of C36/LJ-PME agree well with the OpenMM ones, except very close to phase transition temperatures, where even subtle differences in the implementation of the used algorithms can lead to qualitatively different behavior.

**Figure 1:**
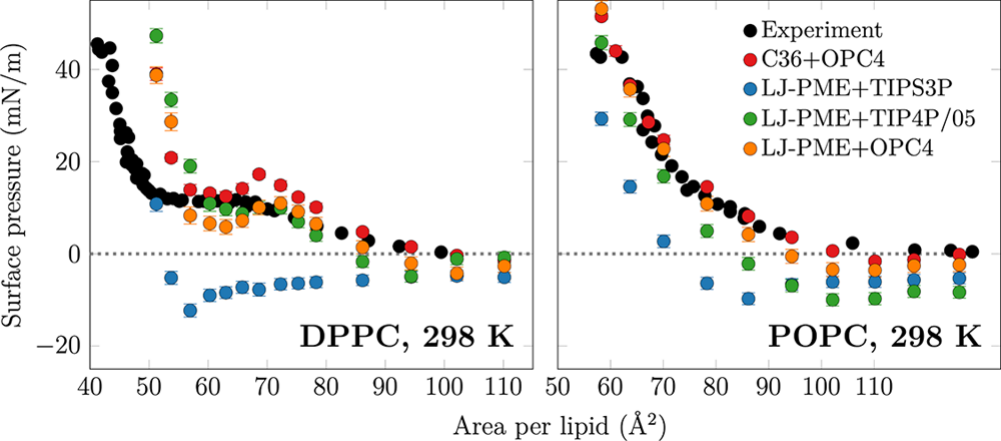
Surface pressure–area isotherms for DPPC and POPC at 298 K obtained with the C36/LJ-PME lipid model (LJ-PME) and with different water models in this work. Additionally, data for the standard C36 simulated with OPC4 water, taken from our earlier work,^15^ are shown together with experimental data extracted from well-equilibrated monolayers.^49^

**Figure 2:**
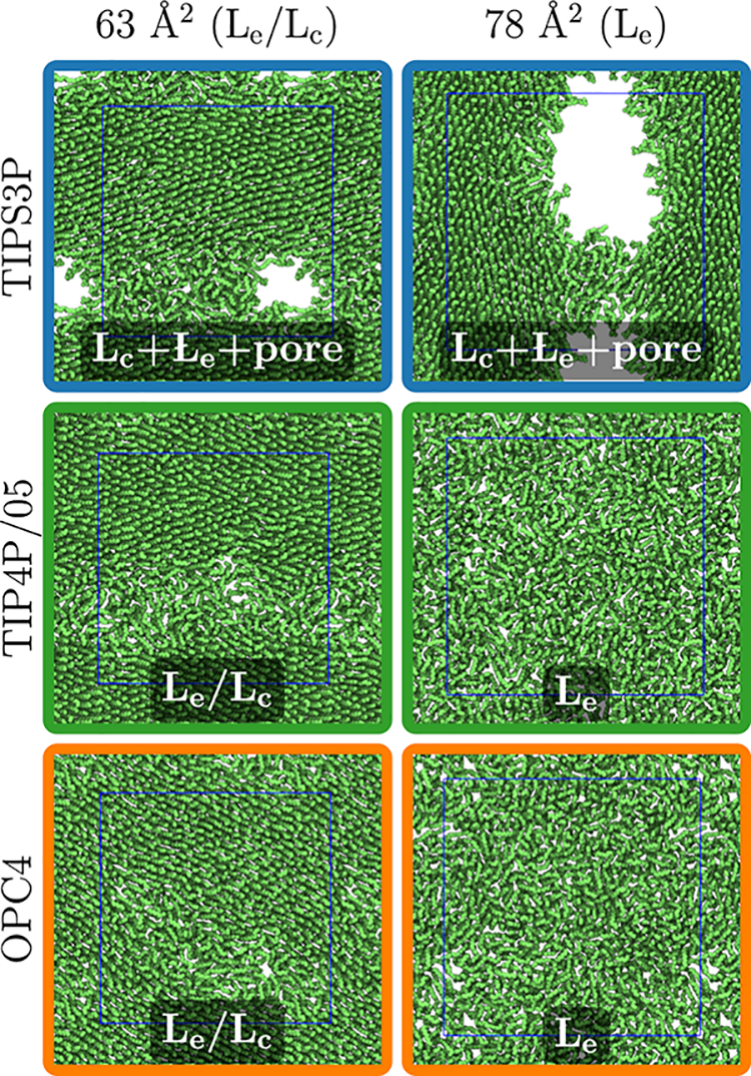
Snapshots of the DPPC monolayer at two APL values from the C36/LJ-PME lipid model with three different water models. The labels on top indicate the expected phases based on experiments, whereas those on the snapshots show the observed ones with the used model.

To test the performance of C36/LJ-PME in monolayer simulations, we compared the surface pressure–area isotherms of DPPC and POPC monolayers with the isotherms from standard C36 with OPC4 water from our previous work^15^ and experiments^49^ in Figure 1. Both systems are simulated at 298 K which is well below the *T*_m_ of DPPC yet well above the *T*_m_ of POPC, therefore ensuring that we are not close to any phase transitions. The C36/LJ-PME with the TIPS3P water model suffers from characteristic issues for monolayer simulations performed with water models having too low surface tension: Negative surface pressures, corresponding to nonphysical states where the absorbance of a surfactant layer *increases* the interfacial tension, appear above an APL of 51 Å^2^ for DPPC and 70 Å^2^ for POPC in C36/LJ-PME simulations. Furthermore, stable pores appear in monolayers at an APL of 60 Å^2^ for DPPC (Figure 2) and 86 Å^2^ for POPC, which are significantly below the experimental values where the gas–L_e_ phase coexistence begins; approximately 100–110 and 120–130 Å^2^, respectively.^49,50^ The opening of pores at too small APLs can be explained by the too low surface tension of the TIPS3P water model favoring the exposure of water surface rather than the transition of most lipids to the L_e_ phase upon increasing APL. Notably, such pores may not appear in simulations with small box size due to finite size effects,^14^ which could be the case in monolayer simulations with 36 lipids used in the optimization protocol of the C36/LJ-PME model.^17^

**Figure 3:**
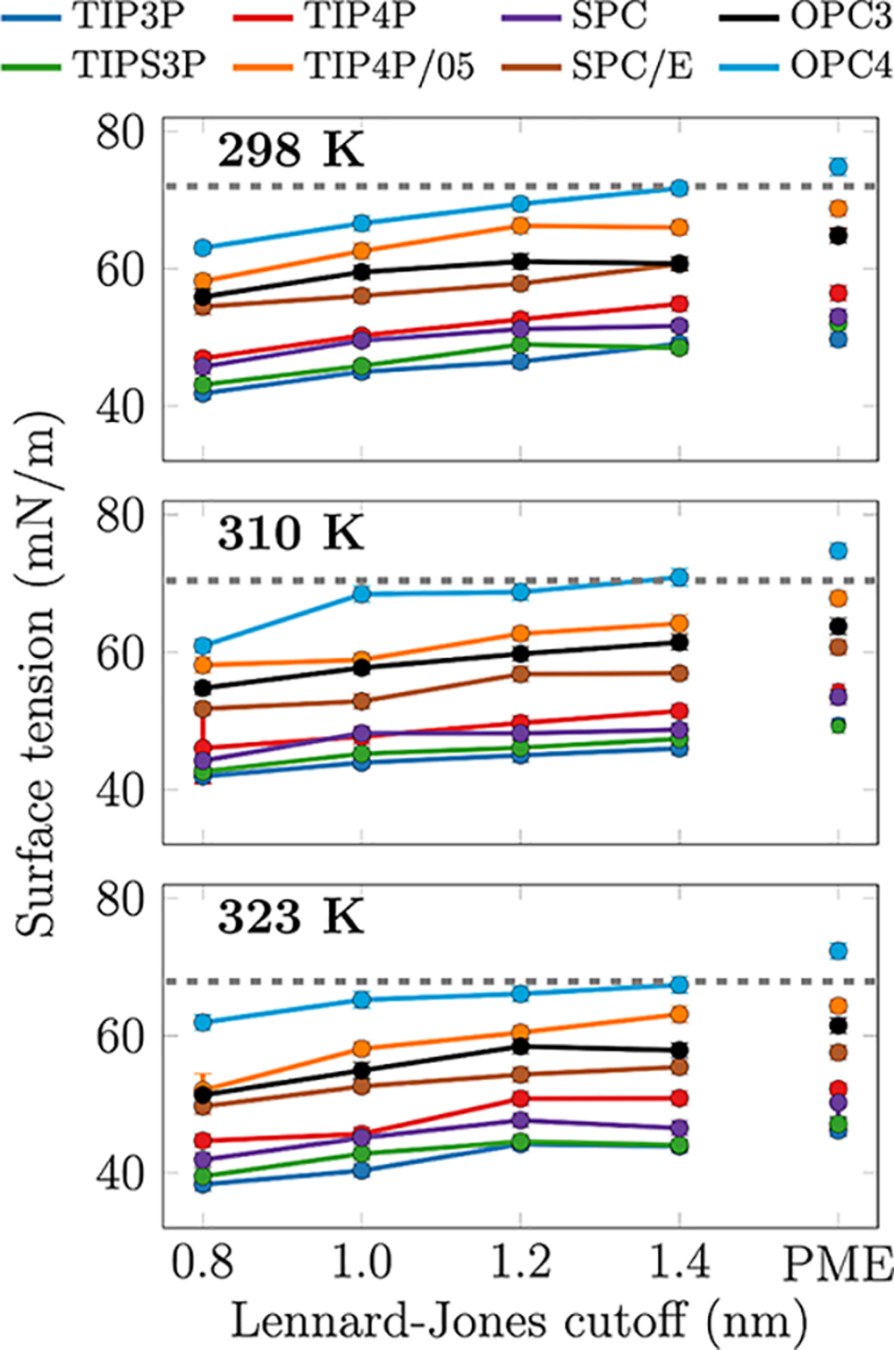
Surface tension of commonly used water models with different LJ cutoffs and at three temperatures.

Because C36/LJ-PME with TIPS3P showed a behavior characteristic for simulations with too low water surface tension, we set out to find a water model that reproduces the experimental surface tension with LJ-PME that could be used together with the C36/LJ-PME lipid model. To this end, we evaluated the surface tension of eight water models at three different temperatures using different Lennard-Jones cutoff distances and Lennard-Jones PME in Figure 3. The numeric values are available in Table S1 in the SI. Our results with LJ-PME agree reasonably well with those extracted at 300 K by Sega and Dellago,^51^ differing on average by ∼2%. The largest deviation is observed for SPC/E, for which our values are ∼3 mN/m larger than those extracted by Sega and Dellago^51^ or by in’t Veld et al.^52^ Water surface tension increases in all models with the increasing cutoff, converging toward the values obtained with LJ-PME as expected. As also shown previously, OPC4 performs reasonably well with cutoffs of 1.2 and 1.4 nm^15^ but slightly overshoots the experimental value with LJ-PME. TIP4P/05 slightly undershoots water surface tension with LJ-PME, whereas other models behave poorly, with TIP3P and TIPS3P underestimating the experimental values by ∼20 mN/m at all studied temperatures.

Based on the results in Figure 3, we repeated the DPPC and POPC bilayer and monolayer simulations using C36/LJ-PME with OPC4 and TIP4P/05 water models that gave the best surface tension values with LJ-PME. Area per lipid values from DPPC and POPC bilayers simulated at different temperatures suggest that the OPC4 water model is well compatible with the C36/LJ-PME parameters, yet the use of TIP4P/05 water resulted in too large APL values, especially for POPC (Figure S1 and the discussion in the SI).

Next, we calculated the surface pressure–area isotherms for DPPC and POPC with these models (Figure 1). Large negative surface pressures were not observed in these simulations, and the monolayer phase behavior was consistent with experiments and the standard C36 model with the OPC4 water (Figure S2 in the SI). Instead of pore formation at too low areas, the L_e_/L_c_ coexistence was observed for DPPC, as demonstrated in Figure 2 between areas per lipid of 57 and 75 Å^2^. The surface pressure of the coexistence plateau was captured by both OPC4 and TIP4P/05. However, in the L_e_ region of DPPC with APLs above 75 Å^2^, OPC4 and TIP4P/05 undershot the isotherms from experiments. Notably, the combination of the standard LJ cutoff-based C36 model and the OPC4 water model also performed well in this region.^15^ For the POPC monolayer, simulations with TIP4P/05 give too low surface pressure at all APLs.

**Figure 4:**
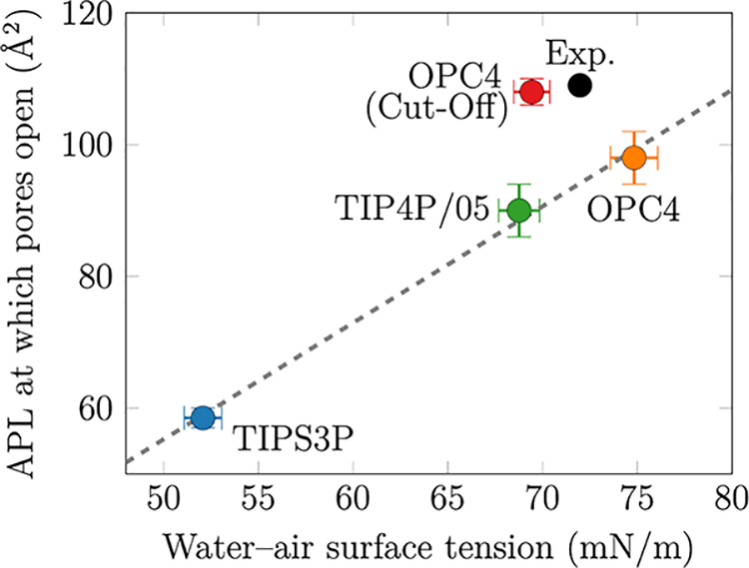
Dependence of the APL of pore formation in the DPPC monolayer on the surface tension of the used water model. The points simulated with C36/LJ-PME fall on a line that does not cross the experimental data point,^50,54^ whereas the simulation with the standard C36 lipids, OPC4 water, and LJ cutoff falls close to the experimental data point.

Depending on the rate of compression, the experimental surface pressure–area isotherms can greatly vary in their shape and positioning.^12^ However, this issue is more critical for small APLs, whereas the behavior of more expanded monolayers is independent of the compression rate.^53^ Thus, we further evaluate our simulation models by analyzing the APL values where pores begin to form in DPPC and compare these to the experimental value from vibrational spectroscopy.^50,54^Figure 4 suggests an approximately linear dependence between the pore formation APL and surface tension of water in the simulation. However, the line fitted to the data does not pass through the experimental data point, yet an offset of ∼10 mN/m is observed, suggesting that adjustments to the C36/LJ-PME lipid model are also required to correctly capture the pore formation tension. In contrast, the simulations performed with the standard C36 lipid model and the OPC4 water with LJ cutoff^15^ are in excellent agreement with the experimental data point in Figure 4. The discrepancy in the pore forming APL may originate from the procedure to derive C36/LJ-PME parameters where parameters were fitted to reproduce the monolayer surface tension, γ(APL) in eq 2, at three APL values.^17^ Because the surface tension of the TIPS3P water model, γ_0_ in eq 2, is too low, parameters that reproduce the correct γ(APL) lead to too low surface pressure, Π(APL) in eq 2.

## 4 Conclusions

The inclusion of long-range van der Waals interactions into the C36/LJ-PME model is an important step toward more realistic MD simulations of interfaces, reducing artifacts arising, for example, from acyl chain–vacuum tension.^16,17^ However, C36/LJ-PME together with its standard water model, TIPS3P, fails to reproduce the experimental surface pressure–area isotherms of DPPC and POPC monolayers. Moreover, these monolayers do not reproduce the experimental phase behavior, and pores are observed at significantly too small values of area per lipid. All of these discrepancies can be traced back to the too low surface tension of the TIPS3P water model, and the pores open as the penalty of exposing water surface is too small. Combining the C36/LJ-PME lipid model with the OPC4 water model—whose surface tension is closer to the experimental one—considerably improves the monolayer phase behavior and agreement with experimental surface pressure–area isotherms. However, the pore forming pressure is underestimated by 10 mN/m when compared to experimental estimates.

It seems that the most realistic lipid monolayer simulations can still be performed by combining the OPC4 water and standard C36 lipid model with the cutoff-based LJ treatment.^15,19^ Surprisingly, an inclusion of long-range van der Waals in C36/LJ-PME did not lead to major improvements when compared with experiments, even though a water model with almost correct surface tension was used. A potential reason for this could be the use of the TIPS3P water model, with a surface tension of approximately 20 mN/m too low, in the parametrization of the C36/LJ-PME lipid model. This underestimated water surface tension is balanced by monolayer tension when optimizing against experimental surface tension values, which leads to underestimated surface pressure values. Furthermore, the small monolayers used in the optimization may become trapped in local minima with surface tensions that are very different from their equilibrium values because the formation of pores is prevented by finite size effects.^14^

While the introduction of long-range van der Waals interactions in lipid bilayer and monolayer simulations is highly desirable, we conclude that the correct water surface tension is more critical to reproduce the experimental surface pressure–area isotherms and monolayer phase behavior. On the other hand, an increasing number of studies suggest that properties of the water model are critical in many applications of MD simulations, such as studies of protein dynamics^55^ and conformational ensembles of disordered proteins.^56,57^ In this light, biomolecular force fields would certainly benefit from the steady improvement of water models since their initial release.^58^ However, the possible effects of changing the water model must be evaluated with care because TIPS3P was involved in the original parametrization of the CHARMM force field. A consistent reparametrization of the entire CHARMM force field family other than the TIPS3P water model would be a gargantuan task even with the help of recently introduced automated approaches.^16,17^ Nevertheless, our previous work^15^ demonstrated that OPC4 did not lead to major structural changes in DPPC and POPC bilayers as compared to TIPS3P, suggesting that CHARMM36 could be safely used with OPC4 in lipid monolayer and bilayer simulations. However, the case with proteins seems more complicated as two studies have reached somewhat different conclusions on the effects of changing water models.^59,60^ Interestingly, TIPS3P did not result in the best agreement with experiments in either study.

Furthermore, the lack of electronic polarizability may limit the applicability of models with fixed partial charges in varied environments.^61^ The polarizable CHARMM Drude lipid model^62,63^ is paired with the SWM4-NDP water model with realistic surface tension,^64^ thereby having the potential to also correctly capture lipid monolayer behavior. However, polarizable models require a significant amount of parametrization work, and they are computationally demanding. On the other hand, an implicit inclusion of polarization by the electronic continuum correction (ECC) reduces artifacts arising from missing electronic polarizability^65−67^ and can be readily applied in monolayer simulations,^9^ although its validity at the air–water interface can be questioned.^67^ Therefore, using the state-of-the-art water models possibly with ECC included during the systematic parametrization of force fields would most likely not only improve the description of monolayer behavior but also facilitate other applications of MD simulations.

## Supporting Information Available

Lipid bilayer simulation methods and areas per lipid of the bilayers with different water models; the numeric values of the surface tensions of various water models; phase behavior of DPPC monolayers with a different water model as revealed by clustering analysis (PDF)
