# Supplementary material for: Accurate Simulations of Lipid Monolayers Require a Water Model with Correct Surface Tension
Source: J Chem Theory Comput. 2022 Feb 8;18(3):1862–9. doi: 10.1021/acs.jctc.1c00951 (PMC8908734; doi:10.1021/acs.jctc.1c00951)
Supplement: Supplementary file 1 — ct1c00951_si_001.pdf [file ct1c00951_si_001.pdf]

Supporting Information for: Accurate Simulations of Lipid Monolayers Require a Water Model With Correct Surface Tension

Carmelo Tempra,[†] O.H. Samuli Ollila,[‡] and Matti Javanainen[*,‡,†]

†*Institute of Organic Chemistry and Biochemistry, Czech Academy of Sciences, Flemingovo nám. 542/2, 160 00 Prague 6, Czech Republic*

‡*Institute of Biotechnology, University of Helsinki, 00014 Helsinki, Finland*

E-mail: matti.javanainen@gmail.com

Lipid Bilayer simulations

Methods

We simulated DPPC and POPC bilayers with the C36/LJ-PME lipid model and with three different water models and at 5 different temperatures to both validate our implementation of the C36/LJ-PME model in GROMACS,[1] as well as to study the effect of different water models on the lipid bilayer behavior. DPPC and POPC bilayer simulations were set up to mimic the DPPC bilayer simulations in Refs. 2 and 3 (larger system): Bilayers with a total of 288 lipids and 31 water molecules per lipid were constructed with CHARMM-GUI.[4] We performed the simulations at five temperatures for both lipid types; 323, 328, 333, 338, and 343 K for DPPC and 298, 303, 308, 313, and 318 K for POPC. In addition to the

TIPS3P model used in the original CHARMM36/LJ-PME,[2,3] we repeated the simulations with two additional water models, namely OPC4 and TIP4P/05, since they best described the surface tension of water. We performed the simulations with the simulation parameters suggested for GROMACS and CHARMM36[4] but with Lennard-Jones PME. Namely, these agree with the simulation parameters used for lipid monolayers (see main text), except that the pressures were additionally coupled semi-isotropically to a Parrinello–Rahman barostat[5] with a target pressure of 1 bar, compressibility of 4.5×10^{-5} 1/bar, and a time constant of 5 ps. We also repeated the DPPC simulations with the standard CHARMM36 lipid model without LJ-PME,[6] and for these simulations we only employed the TIPS3P water model. All simulations were 300 ns long, and the first 100 ns was omitted from analyses.

The area per lipid was extracted by calculating the total bilayer area with `gmx energy`, and dividing this by the number of lipids in one leaflet. The standard error was obtained from block averaging performed by `gmx analyze`.

Evaluation of our Implementation of C36/LJ-PME in GROMACS

Overestimated melting points for DPPC and over-condensed lipid bilayers have been reported when the standard C36 force field[6] is used with GROMACS simulation engine.[4,8] Therefore, we carefully evaluate here the performance of our C36/LJ-PME implementation for GROMACS comparing the DPPC and POPC bilayer APLs against previous results and experimental data in different temperatures in Fig. S1.

Our GROMACS implementation is in good agreement with the reported results from C36/LJ-PME simulations with OpenMM for DPPC at 333 K and for POPC at 303 K.[2,3] For POPC, simulation results agree with experimental data,[7] but give slightly too low values for DPPC. However, at 323 K, the DPPC bilayer is in the ripple phase in C36/LJ-PME simulations with GROMACS, thus giving smaller APL than in experiments and OpenMM simulations. This is in line with the overestimated melting points for DPPC in the standard C36 model when simulated with GROMACS.[8]

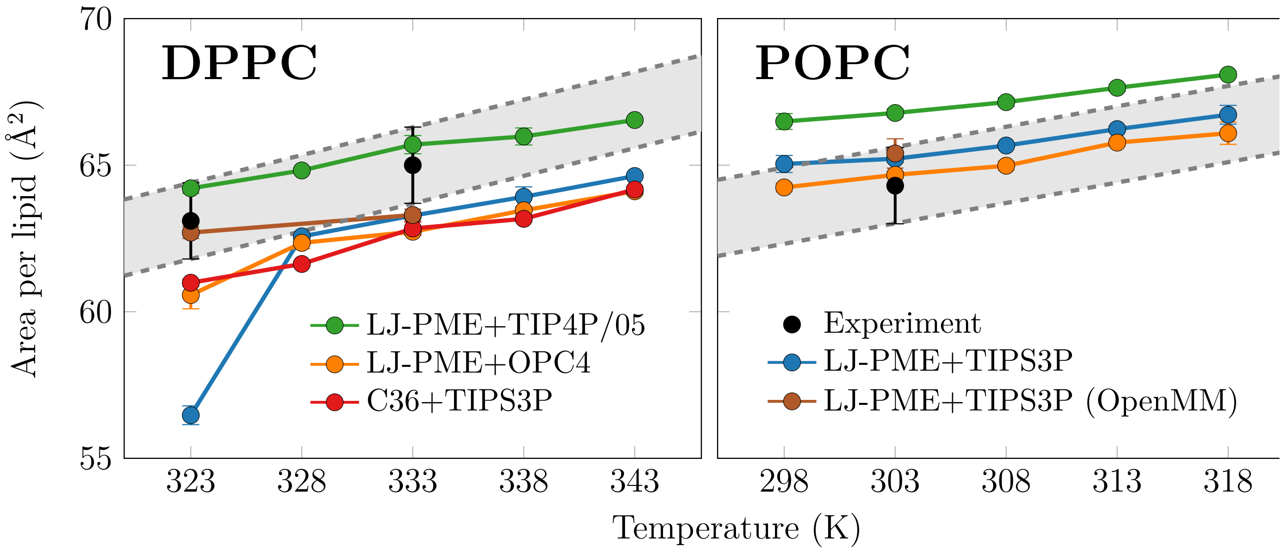

Figure S1: Area per lipid of the DPPC (left) and POPC (right) bilayers simulated at different temperatures with either the standard CHARMM36 (C36) or the C36/LJ-PME (LJ-PME) and with different water models. The black markers and the shaded region show the experimentally determined area per lipid and its extrapolation to other temperatures based on the thermal area expansivity values taken from Ref. 7. The data obtained with CHARMM/OpenMM in Refs. 2 & 3 are shown in brown.

To conclude, our C36/LJ-PME parameters converted to GROMACS format provide the same behavior as OpenMM simulations reported in the literature,[2,3] except close to the liquid–gel transition of DPPC, where small differences in the implementation of the algorithms between simulation engines and different simulation times may dominate the results.

Fortunately, interesting phenomena in DPPC monolayers studied in the main text, such as the L_e/L_c phase coexistence, occur at room temperature which is well below the T_m of DPPC. On the other hand, the T_m of POPC falls below the freezing point of water and is thus not of importance. Therefore, the temperature in our lipid monolayer simulations is sufficiently far from T_m of both lipids to avoid the sensitivity to simulation engine details.

Validation of C36/LJ-PME With Alternative Water Models

The areas per lipid of the DPPC bilayers simulated with the OPC4 and TIP4P/05 water models are shown in orange and green in Fig. S1, respectively. While OPC4 shows great

agreement with experiment and slightly smaller values than the simulations with TIPS3P, the values obtained using TIP4P/05 somewhat overshoot the experimental APL values.

Lipid Monolayer Phase Behaviour

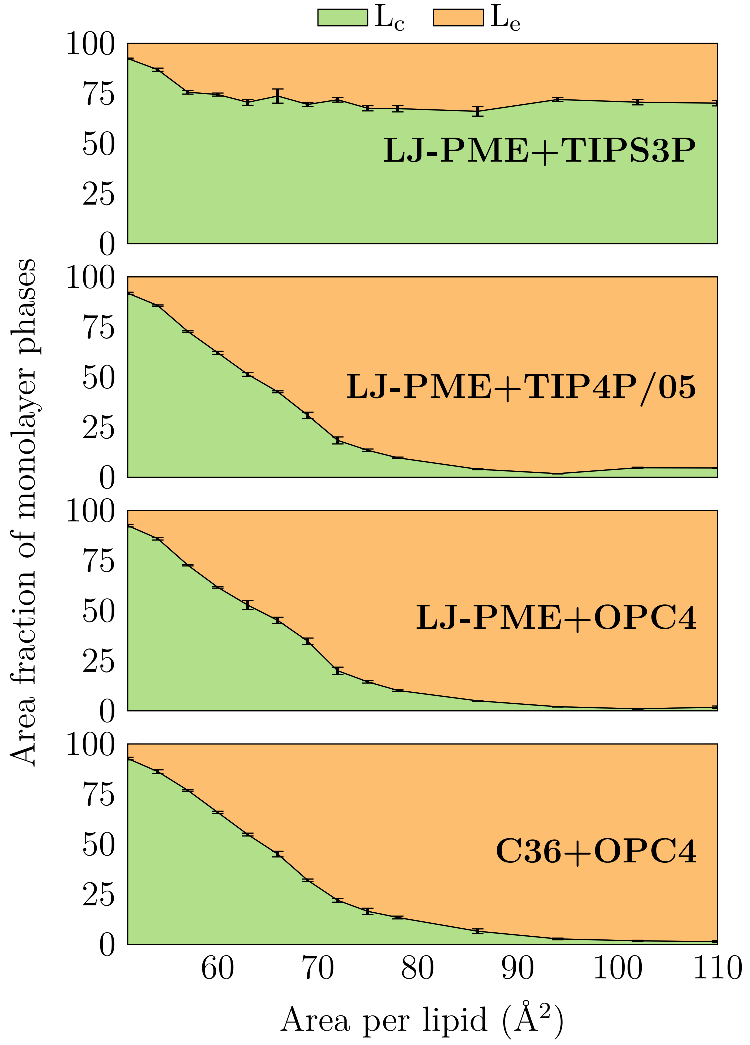

Figure S2: The fractions of chains that show L_c- and L_e-like packing in the DPPC monolayers. Pores are present in the monolayer with TIPS3P at areas of 60 Å^2 and larger, which results in the high L_c-fraction.

Surface Tensions of Water Models

Table S1: Surface tension values for the different water models at different temperatures and with different Lennard-Jones treatment. These data are shown in Fig. 3 in the main text.

**Model**	**Temp.**	**0.8 nm**	**1.0 nm**	**1.2 nm**	**1.4 nm**	**LJ-PME**
TIP3P	298 K	41.8±0.9	45.0±0.9	46.5±1.0	49.1±0.9	49.7±1.0
	310 K	41.9±0.9	43.9±0.8	45.0±1.0	46.0±1.0	49.6±0.8
	323 K	38.3±0.9	40.4±1.0	44.2±0.9	43.9±1.0	46.2±0.9
TIPS3P	298 K	43.1±0.9	45.8±0.7	49.0±0.9	48.5±0.9	52.1±1.0
	310 K	42.6±1.0	45.2±1.0	56.1±1.0	47.4±1.0	49.2±0.9
	323 K	39.5±1.1	42.8±1.0	44.6±1.0	44.1±0.8	47.1±1.1
SPC	298 K	45.7±1.1	49.5±0.8	51.2±0.8	51.7±0.8	53.0±1.0
	310 K	44.2±0.9	48.2±1.0	48.2±0.9	48.7±0.9	53.4±1.1
	323 K	41.9±1.0	45.1±0.9	47.7±1.0	46.5±1.0	50.3±2.3
SPC/E	298 K	54.5±1.1	56.1±1.0	57.8±1.0	60.7±1.0	64.9±1.1
	310 K	51.8±0.9	52.8±1.1	56.8±1.1	56.9±0.9	60.7±1.0
	323 K	49.7±1.1	52.6±0.8	54.3±1.0	55.5±1.0	57.6±1.0
TIP4P	298 K	46.9±0.9	50.3±0.7	52.6±1.0	54.9±1.0	56.5±1.1
	310 K	46.0±5.2	47.7±1.1	49.7±1.0	51.4±0.9	54.4±0.9
	323 K	44.7±0.9	45.7±1.0	50.8±1.0	50.9±1.0	52.2±0.8
TIP4P/05	298 K	58.2±1.0	62.6±1.1	66.3±1.1	66.0±1.1	68.8±1.1
	310 K	58.1±1.1	58.9±1.0	62.7±1.0	64.2±1.2	67.8±1.0
	323 K	52.0±2.4	58.1±1.0	60.5±1.0	63.1±1.2	64.3±0.9
OPC3	298 K	55.9±1.1	59.5±1.0	61.1±1.1	60.8±0.9	64.8±1.0
	310 K	54.8±0.9	57.7±0.9	59.8±1.0	61.4±1.1	63.8±1.2
	323 K	51.4±1.1	54.9±1.2	58.4±1.1	57.9±1.0	61.5±1.1
OPC4	298 K	63.0±0.8	66.6±1.1	69.4±1.0	71.1±1.0	74.8±1.3
	310 K	60.9±1.0	68.4±1.1	68.8±1.2	70.9±1.2	74.8±1.1
	323 K	61.9±1.1	65.2±1.2	66.1±1.1	67.4±1.2	72.3±1.1

References

(1) Páll, S.; Zhmurov, A.; Bauer, P.; Abraham, M.; Lundborg, M.; Gray, A.; Hess, B.; Lindahl, E. Heterogeneous parallelization and acceleration of molecular dynamics simulations in GROMACS. *The Journal of Chemical Physics* **2020**, *153*, 134110.

(2) Yu, Y.; Kramer, A.; Venable, R. M.; Simmonett, A. C.; MacKerell Jr, A. D.; Klauda, J. B.; Pastor, R. W.; Brooks, B. R. Semi-automated optimization of the CHARMM36 lipid force field to include explicit treatment of long-range dispersion. *Journal of Chemical Theory and Computation* **2021**, *17*, 1562–1580.

(3) Yu, Y.; Krämer, A.; Venable, R. M.; Brooks, B. R.; Klauda, J. B.; Pastor, R. W. CHARMM36 Lipid Force Field with Explicit Treatment of Long-Range Dispersion: Parametrization and Validation for Phosphatidylethanolamine, Phosphatidylglycerol, and Ether Lipids. *Journal of Chemical Theory and Computation* **2021**, *17*, 1581–1595.

(4) Lee, J.; Cheng, X.; Swails, J. M.; Yeom, M. S.; Eastman, P. K.; Lemkul, J. A.; Wei, S.; Buckner, J.; Jeong, J. C.; Qi, Y., et al. CHARMM-GUI input generator for NAMD, GROMACS, AMBER, OpenMM, and CHARMM/OpenMM simulations using the CHARMM36 additive force field. *Journal of chemical theory and computation* **2016**, *12*, 405–413.

(5) Parrinello, M.; Rahman, A. Polymorphic transitions in single crystals: A new molecular dynamics method. *Journal of Applied physics* **1981**, *52*, 7182–7190.

(6) Klauda, J. B.; Venable, R. M.; Freites, J. A.; O'Connor, J. W.; Tobias, D. J.; Mondragon-Ramirez, C.; Vorobyov, I.; MacKerell Jr, A. D.; Pastor, R. W. Update of the CHARMM all-atom additive force field for lipids: validation on six lipid types. *The journal of physical chemistry B* **2010**, *114*, 7830–7843.

(7) Kučerka, N.; Nieh, M.-P.; Katsaras, J. Fluid phase lipid areas and bilayer thicknesses

of commonly used phosphatidylcholines as a function of temperature. *Biochimica et Biophysica Acta (BBA)-Biomembranes* **2011**, *1808*, 2761–2771.

(8) Pluhackova, K.; Kirsch, S. A.; Han, J.; Sun, L.; Jiang, Z.; Unruh, T.; Böckmann, R. A. A critical comparison of biomembrane force fields: structure and dynamics of model DMPC, POPC, and POPE bilayers. *The Journal of Physical Chemistry B* **2016**, *120*, 3888–3903.
